# Alginate Oligosaccharides Alleviate Salt Stress in Rice Seedlings by Regulating Cell Wall Metabolism to Maintain Cell Wall Structure and Improve Lodging Resistance

**DOI:** 10.3390/plants13091215

**Published:** 2024-04-28

**Authors:** Youwei Du, Huimin Zhao, Naijie Feng, Dianfeng Zheng, Aaqil Khan, Hang Zhou, Peng Deng, Yaxing Wang, Xutong Lu, Wenxin Jiang

**Affiliations:** 1 College of Coastal Agriculture Sciences, Guangdong Ocean University, Zhanjiang 524088, China; youweidu2023@163.com (Y.D.); ahuiminzhao@163.com (H.Z.); aaqil_agron@hotmail.com (A.K.); zjh1798@163.com (H.Z.); dpylly@163.com (P.D.); yaxinwang2021@163.com (Y.W.); lluuxxutt@163.com (X.L.); jwenzi21@163.com (W.J.); 2 South China Center of National Saline-Tolerant Rice Technology Innovation Center, Zhanjiang 524088, China; 3 Shenzhen Research Institute of Guangdong Ocean University, Shenzhen 518108, China

**Keywords:** rice, salt, alginate oligosaccharides, cell wall, cell wall metabolism, lodging-resistant index

## Abstract

Salt stress is one of the major abiotic stresses that damage the structure and composition of cell walls. Alginate oligosaccharides (AOS) have been advocated to significantly improve plant stress tolerance. The metabolic mechanism by which AOS induces salt tolerance in rice cell walls remains unclear. Here, we report the impact of AOS foliar application on the cell wall composition of rice seedlings using the salt-tolerant rice variety FL478 and the salt-sensitive variety IR29. Data revealed that salt stress decreased biomass, stem basal width, stem breaking strength, and lodging resistance; however, it increased cell wall thickness. In leaves, exogenous AOS up-regulated the expression level of *OSCESA8*, increased abscisic acid (ABA) and brassinosteroids (BR) content, and increased β-galacturonic activity, polygalacturonase activity, xylanase activity, laccase activity, biomass, and cellulose content. Moreover, AOS down-regulated the expression levels of *OSMYB46* and *OSIRX10* and decreased cell wall hemicellulose, pectin, and lignin content to maintain cell wall stability under salt stress. In stems, AOS increased phenylalamine ammonia-lyase and tyrosine ammonia-lyase activities, while decreasing cellulase, laccase, and β-glucanase activities. Furthermore, AOS improved the biomass and stem basal width and also enhanced the cellulose, pectin, and lignin content of the stem, As a result, increased resistance to stem breakage strength and alleviated salt stress-induced damage, thus enhancing the lodging resistance. Under salt stress, AOS regulates phytohormones and modifies cellulose, hemicellulose, lignin, and pectin metabolism to maintain cell wall structure and improve stem resistance to lodging. This study aims to alleviate salt stress damage to rice cell walls, enhance resistance to lodging, and improve salt tolerance in rice by exogenous application of AOS.

## 1. Introduction

Rice (*Oryza sativa* L.) is one of the staple foods consumed by half of the globe’s population and contributes around 30–80% of the daily essential calories [1]. It is a glycophytic plant that is relatively salt-sensitive in early vegetative stages [2]. Salt stress typically causes high osmotic stress, ionic stress, and oxidative stress, which ultimately inhibits normal plant growth and development [3]. Plants are sessile and thus have to establish appropriate mechanisms to mitigate the adverse effects of salt stress [4].

The cell wall is a complex and dynamic structure in plant cells that provides mechanical support and acts as a sensor between the plant and its environmental changes [5]. The composition of plant cell walls includes pectin, cellulose, hemicellulose, lignin, and a minute quantity of protein [6]. And it plays a crucial role in the plant’s response to salt stress [7]. Salt stress leads to various physiological changes such as an increase in the homogalacturonan content, methyl esterification, and modification of hemicellulose structural domains in the polysaccharides of *Artemisia* [8]. Salt stress induces changes in *Chara corallina* cell wall by causing the physical binding of pectin to Na^+^ [9]. Under salt stress, *Lotus tenuis* leaves exhibited a lower cell wall fiber content while producing more hemicellulose and pectin, resulting in a more open cell wall structure [10]. Previous studies focused on specific genes and polyomic cell wall changes induced by salt stress [10]. *SWO1* functions together with importin α to regulate the expression of cell wall-related genes, which enables plants to maintain cell wall integrity under high salinity [11]. The *RRES1* mutant regulates the maintenance of cell wall integrity under salt stress by reducing arabinose and xylose contents [12]. Mutations in the *FC19* gene greatly impact the hemicellulose xylan biosynthesis to improve plant resistance to chlorosis and cadmium [13]. The proteins related by signal transduction and adaptation to cell wall polysaccharides were enhanced in both salt-tolerant tomato IL8-3 and salt-sensitive tomato M82 cell walls in response to salinity stress [14]. In *Arabidopsis*, seven DEGs associated with the synthesis of lignin and 24 DEGs encode plant cell wall proteins, indicating the significance of cell wall restoration under salt acclimation [15]. Research on salt stress has predominantly concentrated on protoplasts, with limited exploration of cell wall metabolic synthesis.

Alginate oligosaccharides (AOS) are degradation products of alginate extracted from brown algae and, due to good biological activities, AOS possesses anti-inflammatory, antimicrobial, and antioxidant properties [16]. AOS regulated the pectin, cellulose, and hemicellulose content of strawberry cell walls [17]. Our previous study demonstrated that alginate oligosaccharides specifically activated genes related to cell walls in rice under salt stress [18]. The regulatory mechanism of alginate oligosaccharides on rice cell wall metabolism under salt stress remains unclear. Plant stem strength is closely related to the amount of non-structural and structural carbohydrates, such as lignin and cellulose content [19]. Plant growth regulators can enhance the mechanical strength of rice plants and improve resistance to lodging [20]. Therefore, we speculated that alginate oligosaccharides could improve rice resistance to salt stress by modulating the cell wall.

Previous studies demonstrated that the extrinsic application of plant growth regulators enhances plant stress tolerance by regulating cell wall compositions. The current study explored the role of AOS in regulating cell wall compositions and the relationship between AOS and the cell wall composition resistance to lodging under salt stress using two rice varieties, i.e., FL478 (salt tolerant) and IR29 (salt-sensitive). The current findings will further enrich our understanding of the physiological mechanisms of salt tolerance and insights to mitigate salt stress in rice.

## 2. Results

### 2.1. Effect of Salt and Alginate Oligosaccharides on Rice Biomass

The effects of salt and alginate oligosaccharide treatments on rice seedlings biomass are shown in (Figure S1). Salt stress significantly reduced the morphological traits of both varieties. There were corresponding percent decreases of 23.13% and 36.3% in fresh weight of leaves, 11.24% and 15.62% in fresh weight of stems, 8.06% and 17.71% in dry weight of leaves, 11.96% and 19.53% in dry weight of stems, respectively for FL478 and IR29. The fresh weight of leaves, stems, and dry weight of stems of both varieties are significantly affected by AOS. Compared with salt stress, AOS treatments increased the percentage to 13.83% and 5.15% in fresh weight of leaves, 10.28% and 3.24% in fresh weight of stems, 4.39% and 6.33% in dry weight of leaves, and 9.56% and 6.80% in dry weight of stems, respectively, for FL478 and IR29. This indicates that salt stress injury was attenuated by alginate oligosaccharide treatment.

### 2.2. Effect of Salt and Alginate Oligosaccharides on Major Components of the Rice Cell Wall

**Figure 1:**
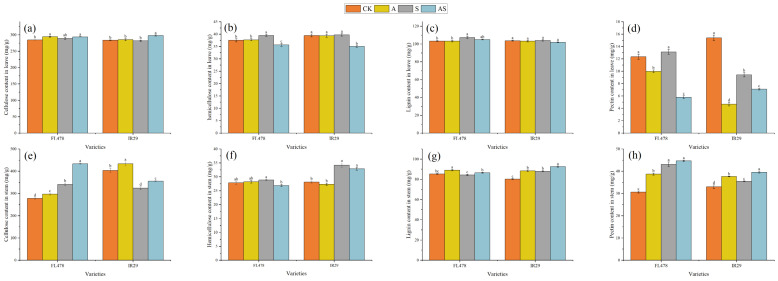
Effect of alginate oligosaccharides and salt on major components of rice cell walls. Leaf cellulose content (**a**), leaf hemicellulose content (**b**), leaf lignin content (**c**), leaf pectin content (**d**), stem cellulose content (**e**), stem hemicellulose content (**f**), stem lignin content (**g**), and stem pectin content (**h**). Control (CK), alginate oligosaccharides (A), salt (S), and alginate oligosaccharides and salt (AS). Different letters in the same variety indicate significant differences between the treatments (*p* < 0.05).

The alginate oligosaccharides and salt treatments had a significant effect on the cellulose, hemicellulose, pectin, and lignin content of the rice leaf and stem cell walls (Figure 1). The major components of the cell wall in different parts of the two rice varieties were affected by salt stress to various degrees. Compared to CK, salt stress significantly the increased pectin content (6.51%) of variety FL478 and decreased the pectin content (38.82%) of variety IR29. There were corresponding percent increases of 3.9% and 0.31% in lignin and 5.35% and 0.68% in hemicellulose content in the leaves of FL478 and IR29, respectively, with the effect reaching significant levels for FL478. Alginate oligosaccharide treatment significantly reduced components by 55.92% and 24.55% in pectin, 9.77% and 11.85% in hemicellulose content, and 2.15% and 2.08% in lignin content, respectively, in the leaves of both cultivars. There was a percentage increase of 1.57% and 5.76% in cellulose content for FL478 and IR29, respectively. The cellulose content in leaves of IR29 was significantly affected by alginate oligosaccharide.

Similarly, compared to CK, salt stress markedly increased the pectin content of the stem by 40.61% and 7.42% and the hemicellulose content by 3.68% and 21.65%, respectively, for FL478 and IR29. The hemicellulose content in the stem of variety FL478 was significantly affected by salt stress. Salt stress significantly increased the cellulose content (22.61%) of FL478, while it significantly decreased the cellulose content (19.69%) of IR29 and remarkably increased the lignin content (9.74%) of IR29. Alginate oligosaccharide + NaCl treatment significantly increased the stem cellulose content by 27.29% and 9.59% and the lignin content by 2.66% and 5.28%, respectively, for FL478 and IR29, compared to salt stress. The hemicellulose content of variety FL478 was markedly decreased by AS. AS improved the pectin content by 3.68% and 11.65%, respectively, for FL478 and IR29. The hemicellulose content of variety FL478 was markedly decreased by AS. The corresponding percent decreases were 6.97% and 3.94%, respectively. 

### 2.3. Transmission Electron Microscopy Study of the Effect of Alginate Oligosaccharides on the Cell Wall Structure of Salt-Stressed Leaves

**Figure 2:**
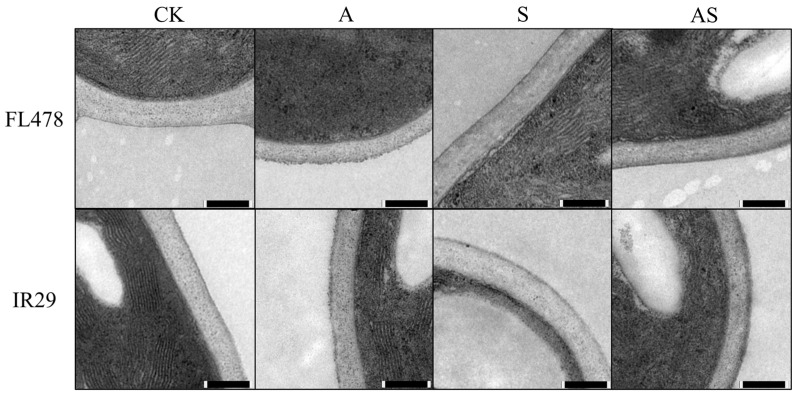
Effect of exogenous alginate oligosaccharide on subcellular structure under salt stress as observed by transmission electron microscopy. Control (CK), alginate oligosaccharides (A), salt (S), and alginate oligosaccharides and salt (AS).

Transmission electron microscopy of the cell wall in rice leaves under salt stress treatment showed thickening of the cell wall in both FL478 and IR29, compared to the control; however, the increase did not reach a statistically significant level. Compared to salt stress, the adverse effects of salt stress on leaf cell walls were alleviated with the addition of alginate oligosaccharide (Figure 2).

### 2.4. Effects of Salt and Alginate Oligosaccharides on Physical Characteristics of Rice Stems, Resistance Index, and Stem Basal Width

**Figure 3:**
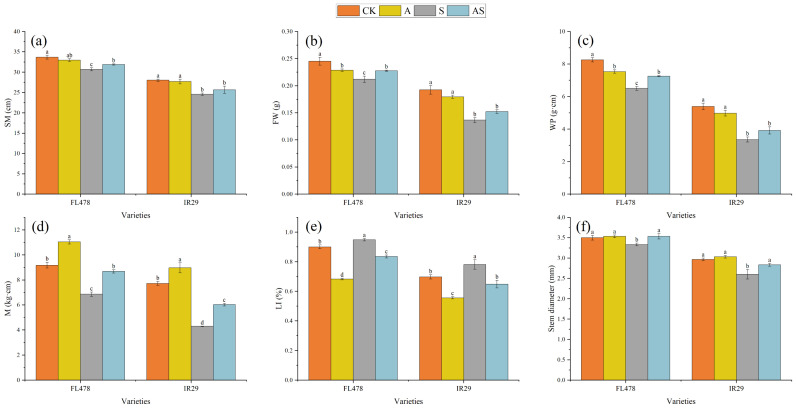
Effects of alginate oligosaccharides and salts on physical characteristics of rice stems, lodging resistance index, and stem basal width. SM (**a**), FW (**b**), WP (**c**), M (**d**), Li (**e**), and stem diameter (**f**). Control (CK), alginate oligosaccharides (A), salt (S), and alginate oligosaccharides and salt (AS). Different letters in the same variety indicate significant differences between the treatments (*p* < 0.05).

Stem cell wall compositions were significantly influenced by salt and alginate oligosaccharides treatments (Figure 3). Salt stress significantly reduced the stem structural strength of both varieties. There were corresponding percent increases of 5.32% and 12.09% in lodging index (LI), while there were significant decreases of 8.90% and 12.37% in the cross-section modulus (SM), 21.27% and 37.70% in the bending moment of the whole plant (WP), 25.27% and 44.40% in the breaking strength (M), and 4.76% and 12.36% in the stem basal width, respectively, for FL478 and IR29. Alginate oligosaccharide + salt treatment significantly increased the percentage as 6.00% and 8.97% in the stem basal width and as 26.67% and 40.30% in M, respectively, for FL478 and IR29. The SM, the fresh weight from the broken point to the panicle top (FW), and the WP of variety FL478 were significantly affected by AOS. Compared to salt stress, AOS treatments increased the percentage by 3.91% and 4.48% in the cross-section modulus, by 7.36% and 11.47% in FW, and by 11.62% and 16.55% in WP, while significantly reducing LI by 11.88% and 17.02%, respectively, for FL478 and IR29. This demonstrates the ability of alginate oligosaccharides to increase rice stem strength and reduce the detrimental effects of salt stress on rice resistance to stunting.

### 2.5. Effects of Salt and Alginate Oligosaccharides on Cellulose-Related Metabolic Enzymes in Rice

The effects of salt and alginate oligosaccharide treatment on cellulose metabolism are shown in (Figure S2). Salt stress significantly reduced the cellulase activity of both varieties. There were corresponding percentages of 14.54%% and 8.53% in leaf cellulase activity and 5.07 and 24.78% in stems cellulase activity, respectively, for FL478 and IR29. Compared with salt stress, alginate oligosaccharide + salt treatment significantly increased the percentage of 18.01% and 44.74% in cellulase activity, respectively, for FL478 and IR29, while significantly decreasing the stem cellulase activity (5.91%) of FL478 and significantly increasing the stem cellulase activity (8.68%) of IR29.

Compared to CK, salt stress remarkably increased the β-glucanase activity by 13.11% and 1.69%, respectively, in leaves and stems for FL478, while significantly decreasing the percentage by 22.60% and 27.56% in β-glucanase activity, respectively, in leaves and stems of variety IR29. Compared to salt stress, alginate oligosaccharide + salt treatment significantly increased leaf β-glucanase activity by 9.29% and 20.91%. AOS significantly increased the stems’ β-glucanase activity (8.34%) of variety IR29 and decreased the stems’ β-glucanase activity (11.89%) of variety FL478.

### 2.6. Effects of Salt and Alginate Oligosaccharides on Rice Hemicellulose-Related Metabolic Enzymes

The alginate oligosaccharides treatment had no significant effect on the xylanase activity (Figure S3). Salt stress reduced the xylanase activity of both varieties. There were corresponding percent decreases of 1.62% and 1.96% in leaves’ xylanase activity and 0.36% and 10.01% in stems’ xylanase activity, respectively, for FL478 and IR29. Compared to salt stress, AOS treatments significantly increased the percentage as 4.96% and 7.66% in xylanase activity, respectively, in leaves and stems of IR29. There was no significant effect on FL478.

### 2.7. Effects of Salt and Alginate Oligosaccharides on Pectin-Related Metabolic Enzymes in Rice

The effects of alginate oligosaccharides and salt on pectin metabolism were investigated (Figure S4). Salt stress significantly increased the pectin metabolism of both varieties. There were corresponding percent increases of 21.30% and 9.27% in leaf β-galactosidase activity, while there were decreases in the stems’ β-galactosidase activity (10.59%) of IR29. In FL478, there was an increase in stems’ β-galactosidase activity at 7.90% of FL478. Compared to salt stress, AOS treatment increased leaves’ β-galactosidase activity by 8.06% and 17.72% and stems’ β-galactosidase activity by 2.40% and 13.49%, respectively, for FL478 and IR29. AOS does not significantly affect the β-galactosidase activity in leaves of variety FL478.

Compared to the control, alginate oligosaccharides increased the polygalacturonase activity of leaves by 1.70% and 3.56%, respectively, for FL478 and IR29. Salt stress significantly reduced leaves’ polygalacturonase activity by 10.19% and 2.14% and stems polygalacturonase activity by 1.80% and 9.58%, respectively, for FL478 and IR29. The polygalacturonase activity in the leaves of FL478 and in the stems of IR29 were significantly affected by salt stress, compared to salt stress, AS treatment increased the activity (16.03%) of FL478. The corresponding percentage increases of the significantly increased stems’ polygalacturonase activity were 7.17% and 14.56%, respectively, for FL478 and IR29.

### 2.8. Effects of Salt and Alginate Oligosaccharides on Lignin-Related Metabolic Enzymes in Rice

**Figure 4:**
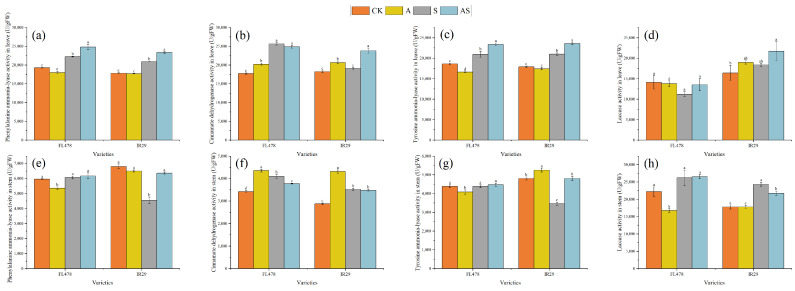
Effect of alginate oligosaccharides and salts on enzyme activities of rice lignin metabolism. Leaf phenylalamine ammonia-lyase activity (**a**), leaf cinnamate dehydrogenase activity (**b**), leaf tyrosine ammonia-lyase activity (**c**), leaf laccase activity (**d**), stem phenylalamine ammonia-lyase activity (**e**), stem cinnamate dehydrogenase activity (**f**), stem tyrosine ammonia-lyase activity (**g**), and stem laccase activity (**h**). Control (CK), alginate oligosaccharides (A), salt (S), and alginate oligosaccharides and salt (AS). Different letters in the same variety indicate significant differences between the treatments (*p* < 0.05).

The effect of salt and alginate oligosaccharide treatments on rice seedling lignin metabolism is shown in (Figure 4). Salt stress significantly increased the leaves’ phenylalamine ammonia-lyase of both varieties. There were corresponding percent increases of 15.36% and 17.69% in phenylalamine ammonia-lyase, respectively, for FL478 and IR29. Salt stress significantly decreased the stems’ phenylalamine ammonia-lyase activity (33.04%) of IR29. Compared to salt stress, alginate oligosaccharide treatment significantly increased the phenylalamine ammonia-lyase activity by 11.56% and 11.19% and stems’ phenylalamine ammonia-lyase activity by 1.77% and 39.75%, respectively, in the leaves of both cultivars. The stems’ phenylalamine ammonia-lyase activity of variety IR29 was not significantly affected by AS.

Compared to the CK, alginate oligosaccharide treatment significantly increased the cinnamic acid dehydrogenase activity of leaves by 13.95% and 13.12% and cinnamic acid dehydrogenase activity of stems by 27.25% and 49.92%, respectively, for FL478 and IR29. Salt stress treatment increased leaves’ cinnamic acid dehydrogenase activity by 44.62% and 4.31% and stems’ cinnamic acid dehydrogenase activity by 19.80% and 22.37%, respectively, for FL478 and IR29. The leaves’ cinnamic acid dehydrogenase activity of IR29 was not significantly affected by salt. AS treatment significantly increased leaves’ cinnamic acid dehydrogenase activity (22.10%) of IR29. There were corresponding percent decreases of 7.85% and 1.23% in stems’ cinnamic acid dehydrogenase activity, respectively, for FL478 and IR29. Salt stress significantly affected the cinnamic acid dehydrogenase activity in stems of variety FL478.

Compared to the control, salt stress significantly increased leaves’ tyrosine ammonia-lyase activity by 11.56% and 17.45%, respectively, for FL478 and IR29, while it decreased stems’ tyrosine ammonia-lyase activity by 28.13% of IR29. Alginate oligosaccharide treatment significantly increased the components by 11.44% and 12.29% in leaves’ tyrosine ammonia-lyase activity and 2.05% and 39.13% in stems’ tyrosine ammonia-lyase activity, respectively, for FL478 and IR29. AOS treatment did not significantly affect the tyrosine ammonia-lyase activity in stems of variety FL478.

Salt stress significantly increased the leaves’ laccase activity (11.87%) of variety IR29. There were corresponding percent increases of 17.90% and 36.93% in stems’ laccase activity, respectively, for FL478 and IR29. Salt stress significantly affected stems’ laccase activity of variety IR29. AOS treatment significantly increased laccase activity by 20.59% and 18.20%, respectively, in the leaves of both cultivars, while it significantly decreased stems’ laccase activity by 10.68% of IR29.

### 2.9. Effect of Salt and Alginate Oligosaccharides on Rice Hormones

**Figure 5:**
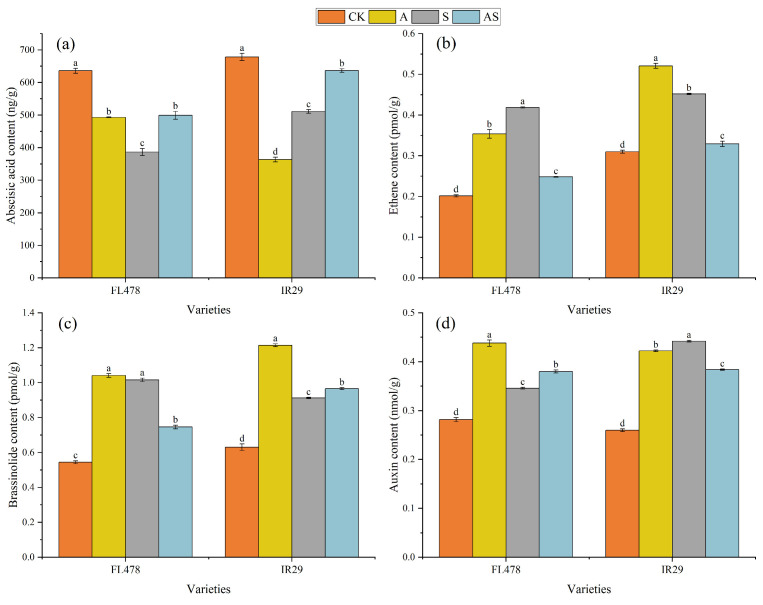
Effect of alginate oligosaccharides and salts on hormone content in rice. Abscisic acid content (**a**), ethylene content (**b**), oleoresin lactone content (**c**), and growth hormone content (**d**). Control (CK), alginate oligosaccharides (A), salt (S), and alginate oligosaccharides and salt (AS). Different letters in the same variety indicate significant differences between the treatments (*p* < 0.05).

The alginate oligosaccharides and salt treatments had a significant effect on the ABA, ETH, BR, and IAA content of the rice leaf (Figure 5). Compared to CK, alginate oligosaccharides significantly reduced by 22.51% and 46.39% in terms of ABA content, while increasing by 91.18% and 92.51% in terms of BR content and by 55.59% and 62.44% with respect to IAA content. Salt treatment significantly decreased by 39.22% and 24.63% in ABA content, while increasing by 107.79% and 45.73% in ETH content, 86.68% and 44.57% in BR content, and 22.89% and 70.05% in IAA content, respectively, for FL478 and IR29. In comparison to salt stress, alginate oligosaccharides significantly increased ABA content by 29.14% and 24.56%, respectively, for FL478 and IR29. Alginate oligosaccharides increased the BR content (5.91%) of variety IR29 and increased the IAA content (9.84%) of variety FL478, while decreasing by 40.73% and 27.14% in ETH content, respectively, for both varieties.

### 2.10. Effect of Salt and Alginate Oligosaccharides on RT-qPCR in Rice

**Figure 6:**
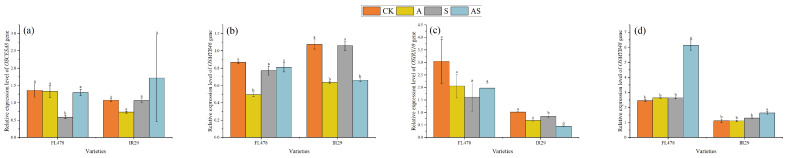
Effect of alginate oligosaccharides and salt on RT-qPCR of rice hormones. *OSCESA8* relative expression level (**a**), *OSMYB46* relative expression level (**b**), *OSIRX10* relative expression level (**c**), and *OSMYB48* Relative expression levels (**d**). Control (CK), alginate oligosaccharides (A), salt (S), and alginate oligosaccharides and salt (AS). Different letters in the same variety indicate significant differences between the treatments (*p* < 0.05).

The effects of salt and alginate oligosaccharide treatments on rice seedlings leaves RT-qPCR are shown in (Figure 6). Compared to salt, AOS treatment significantly upregulated the *OSCESA8* expression levels of both varieties. There were corresponding percent percentage upregulations of 122.54% and 60.93% in *OSCESA8* expression levels, respectively, for FL478 and IR29. The *OSCESA8* expression levels of FL478 are significantly affected by AOS. There were significant corresponding percentage downregulations of 37.54% in *OSMYB46* and 46.88% in *OSIRX10* for IR29. AOS did not significantly affect these two genes of FL478. Sodium alginate significantly upregulated the *OSMYB48* expression levels by 133.04% and 26.40%, respectively, for FL478 and IR29.

## 3. Discussion

The cell wall serves as the primary defense against salt stress [21]. Alginate oligosaccharides have the potential to regulate cell wall compositions under salt stress. We explored the impact of alginate oligosaccharides on the cell wall compositions of rice under salt stress. Normal plant growth and cell wall components are severely influenced by Na^+^ [22]. Salt stress disrupts cellulose synthesis in plants [23]. ABA increased cellulose concentration [24] and BR enhanced plant fibers to reduce stress injury [25]. Alginate oligosaccharide improved the cellulose content by increasing ABA and BR content (Figure 5a,c). Cellulose biosynthesis can be regulated directly or indirectly by regulating cellulose synthase under salt stress [26]. Alginate oligosaccharides upregulated the expression of the cellulose synthesis gene *OSCESA8* under salt stress (Figure 6a). *OSCESA8* is one of the major genes that regulate cellulose synthesis [27]. This led to an increase in the cellulose content of the leaves (Figure 1a). Beta-glucanase is involved in the breakdown of glucan [28]. Cellulase is a crucial enzyme that hydrolyzes cellulose into smaller sugar molecules [29]. The cellulose content and the activity of β-glucanase significantly increased in the leaves and stems of both varieties. It indicates that β-glucanase is not only involved in the breakdown of cellulose but also in the breakdown of xyloglucan in cellulose [30]. In contrast, cellulase and β-glucanase were significantly reduced in FL478 stems, demonstrating the ability of alginate oligosaccharides to increase cellulose content by decreasing the metabolic levels of cellulolytic enzymes. Increased cell wall cellulose might contribute to biomass [31]. The application of alginate oligosaccharides resulted in an increase in rice biomass, which was attributed to elevated cellulose content.

Hemicellulose is a polysaccharide in the cell wall, mainly composed of xylan skeleton and mannose [32]. It has been reported that hemicellulose is greatly involved in plant response to stress [33,34]. The transcription factor *MYB46* regulates xylan synthesis in secondary cell walls, which affects hemicellulose synthesis [35]. *IRX10* plays a significant role in promoting the extension of xylan backbones [36]. Alginate oligosaccharide down-regulated the expression levels of *OSMYB46* and *OSIRX10* in IR29 under salt stress (Figure 6b,c). *OSMYB46* might reduce the synthesis of hemicelluloses such as xylan by regulating *OSIRX10*. The hemicellulose content in leaves and stems increased under salt stress (Figure 1b,f), which agrees with previous studies in cotton [37]. This reduction in the loss of uronic acid in the cell wall during recovery may result from the reduction in polyuronides under salt stress [38]. Xylanase is a glycoside hydrolase that naturally catalyzes the hydrolysis of xylan [39]. The hemicellulose breakdown was promoted by the increase in xylanase activity and β-glucanase activity in leaves and stems in IR29 (Figure S3). Therefore, under salt stress, alginate oligosaccharide reduced the hemicellulose content.

Pectin is a significant constituent of cell wall polysaccharides and is involved in various plant morph-physiological processes, such as plant growth and development, leaf senescence, plant–pathogen interactions, and abiotic stress responses [40]. The changes in pectin content in leaves under salt stress were not consistent, which might be due to the genetic differences between the two cultivars (Figure 1). Alginate oligosaccharides significantly improved β-galacturonase and polygalacturonase activities in the leaves and stems of both rice varieties (Figure S4). Synergistic degradation of cell wall pectin by β-galacturonases and polygalacturonases [41]. Previous studies are confirmed by the previous studies in kiwis and strawberries treated with AOS [17,42,43]. Increased levels of pectin catabolism contributed to a significant decrease in leaves’ pectin content. Leaf and stem regulation is not completely uniform by the application of AOS. The significant increase in enzyme activity in stems did not coincide with the increase in pectin content in stems. This might be due to increased pectin synthesis in the stem, which is regulated by alginate oligosaccharides to improve salt stress tolerance and withstand resistance [44]. Further studies are required to investigate this issue.

Lignin impregnation of secondary cell walls in vascular plants provides mechanical support, impermeability, and resistance to biodegradation [34]. Increased lignin content enhances the degree of cell lignification [45]. Lignification is essential for plant salt tolerance [46]. The present study showed that AOS treatment increased ABA levels under salt stress (Figure 5). ABA is a signaling molecule regulating salt tolerance in rice [47]. ABA induces increased translocation of *MYB7*, promotes flavonoid synthesis, and enhances lignin synthesis [48]. Phenylalamine ammonia-lyase, tyrosine ammonia-lyase, and cinnamate dehydrogenase are critical enzymes involved in lignin synthesis and play an essential role in stress tolerance of rice plants [49]. Phenylalanine ammonia-lyase, cinnamate dehydrogenase, and tyrosine ammonia-lyase activities were increased in leaves (Figure 4a–c). Phenylalamine ammonia-lyase not only plays a role in lignin synthesis but is also an essential enzyme in the phenylalanine synthesis pathway and an antioxidant and improves plant stress tolerance [50,51]. Alginate oligosaccharides increased enzyme activities to promote the removal of reactive oxygen species, which is important for photosynthesis and energy metabolism in leaves [52]. Thus, phenylalamine ammonia-lyase and cinnamate dehydrogenase may be primarily involved in removing reactive oxygen species. Laccase is one of the main oxidizing enzymes in lignin degradation [53]. The decrease in leaves’ lignin content was attributed to the increase in catabolic enzyme activities such as regulating laccase by alginate oligosaccharides (Figure 4d), which regained lignin to normal levels. Lignin plays a greater role in stem stiffness than in leaves. The effect of alginate oligosaccharides on the regulation of different parts of rice was not entirely consistent. AOS increased phenylalamine ammonia-lyase activity in IR29 (Figure 4e) and increased tyrosine ammonia-lyase activity in both species to promote lignin synthesis (Figure 4g). One of the main reasons for the increase in lignin is the decrease in laccase activity in IR29. Lignin in secondary cell walls can act as a diffusion barrier, limiting the entry of Na^+^ and Cl^−^ into xylem channels [9] and blocking the transport of ionic stress to the leaves. Increased lignin in stems helps to maintain osmotic balance and thickened xylem contributes to salt resistance [54]. Therefore, the regulation of lignin content in stems by alginate oligosaccharides under salt stress might provide a pathway for plant leaves to promote physiological processes such as photosynthesis and limit the diffusion of ionic stress into the leaves. However, this aspect still requires further study.

The complex arrangement of cell wall polymers provides mechanical strength, rigidity, and structural integrity, maintains differential growth during cell division and expansion, and serves as a sensory interface between the plant and its environment [55,56]. Therefore, both stress resistance and stress response require the maintenance of cell wall integrity [57]. Secondary cell wall formation and cellulose and lignin deposition patterns are closely related to plant salt tolerance [58]. In the present study, the cell wall was thickened under salt stress (Figure 2). This is mainly due to the increased loosening damage and synthesis of carbohydrates, including pectin and hemicellulose, as a result of salt stress [37]. The formation of a cross-linked network of pore stems in the cell wall causes the carbohydrate to swell, making the cell wall more susceptible to hydrolytic enzymes [59] and causes disruption of pectin and cellulose [60]. The application of AOS regained the cell wall to a normal condition because AOS reduced the pectin and hemicellulose content, stabilized the cell wall structure, and reduced swelling. Cell walls provide mechanical stiffness to the cell to mitigate swelling changes induced by salt stress [37].

Salt stress inhibits normal growth and morphogenesis in rice [61] and can adversely affect the physical properties of rice stems. The culm resistance index is mainly related to breaking strength, plant height, stem diameter, wall thickness, and fresh weight [62,63]. In the present study, salt stress reduced the cellulose content of stem basal width in both rice varieties, resulting in reduced physical strength (Figure 3d). This suggests that salt stress damages cell structure, inhibits plant morphology, and reduces rice resistance to lodging. Alginate oligosaccharide treatment enhances cellulose and lignin levels. Increased stem-breaking strength, which reduces the index of rice resistance to lodging [13], did not change the stem base width significantly, suggesting that the content of rice stem structural material directly affects stem strength [64]. Salt stress significantly increased the lignin, cellulose, and pectin content and stem base width with alginate oligosaccharide treatment, thereby increasing stem strength. Alginate oligosaccharides increased the breaking strength of rice and reduced the risk of rice lodging by regulating the lignin, cellulose, and pectin contents, as well as the stem base width.

**Figure 7:**
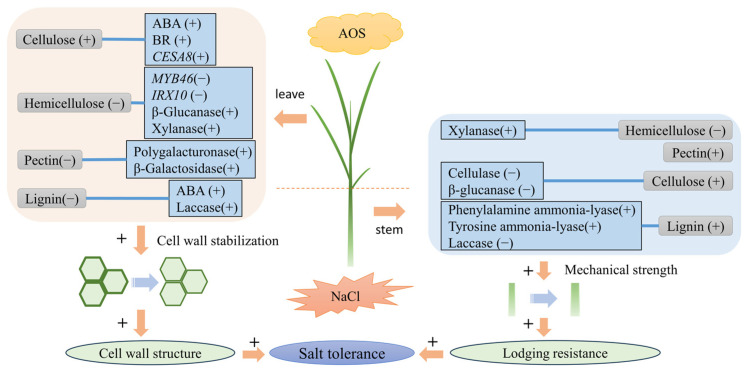
Working model of alginate oligosaccharides regulating rice cell wall metabolism to maintain cell wall stabilization and improve downy mildew resistance under salt stress. (+) and (−) as positive and negative factors. + as an enhanced process.

To understand the role of AOS in maintaining cell wall stabilization and enhancing resistance to lodging under salt stress, a working model is shown (Figure 7). In leaves, AOS up-regulated the expression level of leaf *OSCESA8*, down-regulated the expression level of *OSMYB46* and *OSIRX10*, and increased β-galacturonic, polygalacturonase, xylanase, and laccase activities. It also increased cellulose content and decreased cell wall hemicellulose, pectin, and lignin content to maintain cell wall stability under salt stress. In terms of improving resistance to defoliation, alginate oligosaccharides can do so by modulating a variety of cell wall metabolizing enzymes, including cellulase, laccase, and β-glucanase reduction and by regulating phenylalanine ammonia-lyase and tyrosine ammonia-lyase activities, which increases lignin and cellulose content. Thus, this model highlights the multiple beneficial roles of alginate oligosaccharides in maintaining cell wall stability and improving lodging resistance by regulating rice cell wall metabolism under salt stress.

## 4. Materials and Methods

### 4.1. Plant Material and Test Conditions

The experiment utilized two rice varieties: the salt-tolerant variety FL478 and the salt-sensitive variety IR29. Seeds were acquired from the Germplasm Resource Bank of Coastal Agriculture College at Guangdong Ocean University (Zhanjiang, China). The study was conducted in 2023 at the Coastal Agriculture College’s greenhouse, Guangdong Ocean University (Zhanjiang, China), under controlled conditions (natural light, day/night temperature of 25/20 ± 2 °C, and 60% relative humidity) using potted plants. The nutrient base comprised a 3:1 mixture of red soil and sand, with 2.5 kg per pot (upper base diameter: 210 mm, lower base diameter: 160 mm, height: 180 mm). Seeds were sterilized with 3% H_2_O_2_ for 15 min, rinsed with distilled water, soaked, and germinated for 24 h at 30 °C in the dark before sowing. In total, 800 mL of 1/2 Hoagland nutrient solution was watered on the pot the day before sowing and sowed with 69 seeds per pot. Plants were grown to the 1-leaf-1-heart stage (6 days after sowing) and subjected to simultaneous treatment with NaCl and alginate oligosaccharides (provided by the Dalian Institute of Chemical Physics, Chinese Academy of Sciences). The plants were treated with either distilled water or a solution containing 900 mg·L^−1^ of alginate oligosaccharides (spray the front and back of each leaf evenly to moisten them without causing dripping). Treatments included distilled water + clear water (CK), alginate oligosaccharides + clear water (A), distilled water + NaCl solution (S), and alginate oligosaccharides + NaCl solution (AS). Each treatment had three replicates. The 0.3% NaCl solution or clear water was replenished at 2-day intervals with three times the amount of water to maintain concentration. Samples were collected from four leaves and one heart 15 days after AOS application.

### 4.2. Stem Physical Characterization, Falling Index, and Biomass Measurement

Representative rice samples were taken at the stem base using the YYD-1 instrument (Zhejiang Top Instrument Co., Ltd., Hangzhou, China). The breaking strength was measured at a distance of 2 cm with three replications, each comprising 10 rice plants. Stem physical properties were calculated as follows: whole plant bending moment (WP, g cm) = SL (cm) × FW (g) where SL is the distance from the breaking point to the leaf tip and FW is the fresh weight from the breaking point to the leaf tip. Breaking strength (M, g cm) = 1/4 × BL (kg) × L (cm) where BL is the force to break the stem and L is the distance between the two points. Lodging resistance index Li (%) = WP/M × 100% [20]. Intact rice plants were harvested and leaves and stems were separated to determine their fresh and dry weights.

### 4.3. Extraction of the Cell Wall

Rice tissues were harvested, crushed with liquid nitrogen, homogenized with 75% ethanol for 20 min in ice-cold water, and centrifuged at 10,000 rpm for 10 min, following which the supernatant was removed; this was repeated once and sequentially homogenized and extracted twice with ice acetone, methanol: chloroform (*v*:*v* = 1:1), and methanol. The remaining residue constituted the cell wall material, which was lyophilized and stored at 4 °C for subsequent experiments [65,66].

### 4.4. Determination of Cell Wall Components

Cell walls were lysed with anhydrous ethanol and pectin content was determined by the carbazole method [67]. Lignin content was determined by adding cell wall material to 25% acetyl bromide, neutralizing with sodium hydroxide, and adding hydroxylamine hydrochloride [68]. Cell wall material was added to 2MTFA and, after repeated evaporation with isopropanol, the cellulose content was determined by the anthrone method [67]. Cell wall material was added to concentrated sulfuric acid and hemicellulose content was determined by adding FeCl_3_ and moss black phenol [62].

### 4.5. Determination of Cell Wall-Related Metabolic Enzymes

The extracted enzyme solution was extracted with sodium acetate buffer and the β-glucanase activity was determined by the 3,5-dinitrosalicylic acid method after reacting with β-glucan for 30 min at 37 °C [28]. Determination of xylanase by DNS method after reaction with xylan solution [39]. The enzyme solution was extracted with phosphate buffer, reacted with sodium carboxymethylcellulose, and cellulase (PE) was determined by DNA [69]. Reaction with polygalacturonic acid and DNS assay for polygalacturonase (PG) [70]. Determination of β-galactosidase (β-gal) by reaction with 16 M p-nitrophenyl-β-D-galactopyranoside followed by quenching with sodium carbonate [71]. The enzyme solution was extracted with borate buffer and reacted with L-phenylalanine (PAL) to determine of alanine deaminase [72]. Determination of tyrosine aminotransferase (TAL) was performed using tyrosine [73]. The enzyme solution was extracted with phosphate buffer and reacted with NADP and trans-cinnamic acid to determine cinnamyl alcohol dehydrogenase (CAD) [74]. Laccase (LAC) was determined by extraction of the enzyme solution with acetate buffer and reaction with Syringaladazine [75].

### 4.6. Determination of Hormone Content

ABA, BR, ETH, and IAA were determined using a double antibody one-step sandwich enzyme-linked immunosorbent assay (ELISA) kit. Rice leaves were harvested. Gibberellin (GA) antibody precoated microtiter wells were sequentially coated with sample, standard, and HRP-labeled detection antibody, incubated, and washed thoroughly. The color is developed with the substrate TMB and converted to blue by peroxidase and to its final yellow color in the presence of acid. There is a positive correlation between the color and the concentration of hormones in the sample. The absorbance (OD) was measured at 450 nm using an enzyme labeler and the concentration of the sample was calculated [65].

### 4.7. Transmission Electron Microscopy (TEM) Analysis

Rice leaf cross-sections were prepared to observe the cell wall ultrastructure [13]. Leaf samples were cross-cut (2 mm thick), fixed in 2.5% glutaraldehyde buffer (PBS4), and stored at 4 °C after evacuation. Subsequently, samples were fixed in 0.1 M phosphate buffer PB (pH 7.4) containing 1% osmium acid for 7 h at room temperature without light. Following dehydration in a gradient of anhydrous ethanol and acetone, samples were embedded in acetone and 812 embedding medium for resin sectioning. Sections were stained with 2.6% lead citrate solution for 8 min and observed under TEM (HT7800).

### 4.8. Quantitative Real-Time Fluorescence Assay

Total RNA was extracted using RNAprep Pure (TIANGEN), followed by reverse transcription into cDNA using the PrimeScript kit (TAKRA). qPCR reactions were performed using a LightCycler480II PCR instrument (Roche) and transcript levels were analyzed using the 2^−ΔΔCT^ method [65]. Actin served as the internal reference gene and a detailed list of genes and gene-specific primers is provided in Table S1.

### 4.9. Statistical Analysis

All experiments were conducted with three biological replicates and data are presented as mean ± standard error of the mean (SEM). Statistical analysis, including one-way analysis of variance (ANOVA) and Duncan’s multiple comparison test, was performed using IBM SPSS Statistics 26 (SPSS, Inc., Chicago, FL, USA), with statistical significance set at *p* < 0.05. Digital plots were generated using Origin 2021.

## 5. Conclusions

Salt stress decreased the morphological traits such as dry weight, fresh weight, stem basal width, stem mechanical strength (SM), and resistance index while increasing the leaf cell wall thickness, i.e., leaf hemicellulose and lignin content; however, it increased leaf cell wall thickness and stem pectin content. Exogenous application of alginate oligosaccharides (AOS) explored the ability to regulate hormone levels and alter cellulose, hemicellulose, pectin, and lignin metabolism, thereby enhancing cell wall stability and contributing resistance against lodging in rice. Our findings establish a foundation for understanding the regulatory mechanisms of rice cell walls by AOS under salt stress. Moreover, this study demonstrates a valuable framework and insights for further exploration of the relationship between plant growth regulators and abiotic stress. Future investigations should comprehensively analyze the response of cell wall components and structure to alginate oligosaccharides across the rice life cycle under salt stress to develop a comprehensive understanding of the relationship between cell wall properties and salt stress tolerance.

## Data Availability

The data presented in this study are available on request from the corresponding author.

## Supplementary Materials

The following supporting information can be downloaded at https://www.mdpi.com/article/10.3390/plants13091215/s1. Table S1. Primers used in this study; Figure S1. Effect of alginate oligosaccharides and salt on major components of rice cell wall fresh leaf weight (a), leaf dry weight (b), stem fresh weight (c), and stem dry weight (d); Figure S2. Effect of alginate oligosaccharides and salt on rice cellulase and β-glucanase activities. Leaf β-glucanase activity (a), leaf cellulase activity (b), stem β-glucanase activity (c), and stem cellulase activity (d); Figure S3. Effect of alginate oligosaccharide and salt on rice hemicellulose metabolism. Leaf xylanase activity (a) and stem xylanase activity (b); Figure S4. Effect of alginate oligosaccharides and salts on enzyme activities of rice pectin metabolism. Leaf β-galactosidase activity (a), leaf polygalacturonase (b), stem β-galactosidase activity (c), and stem polygalacturonase (d).
