# Supplementary material for: Alginate Oligosaccharides Alleviate Salt Stress in Rice Seedlings by Regulating Cell Wall Metabolism to Maintain Cell Wall Structure and Improve Lodging Resistance
Source: Plants (Basel). 2024 Apr 28;13(9):1215. doi: 10.3390/plants13091215 (PMC11085217; doi:10.3390/plants13091215)
Supplement: Supplementary file 1 [file plants-13-01215-s001.zip › plants-2964189-supplementary.pdf]

Table S1. Primers used in this study.

gene	Forward（5′- 3′)	Reverse（5′- 3′)
OSCESA8	CTTTGCTAGTATTTGGTTCATCTC	CCACTCCACCTCATCTCA
OSIRX10	GGGAGAATTTCAAGAACAAC	CACAAGCAGAACACAGAT
OSMYB46	GAGGAGGACGAGAAGCTGG	GGGCGGAGGTAGTTGATCC
OSMYB48	TCTTCTTCTTGTTCAGTA	TTCTCATTATTCCTCCTC
actin(nei)	TGAAGTGTGATGTGGATAT	CTCATACGGTCAGCAATA

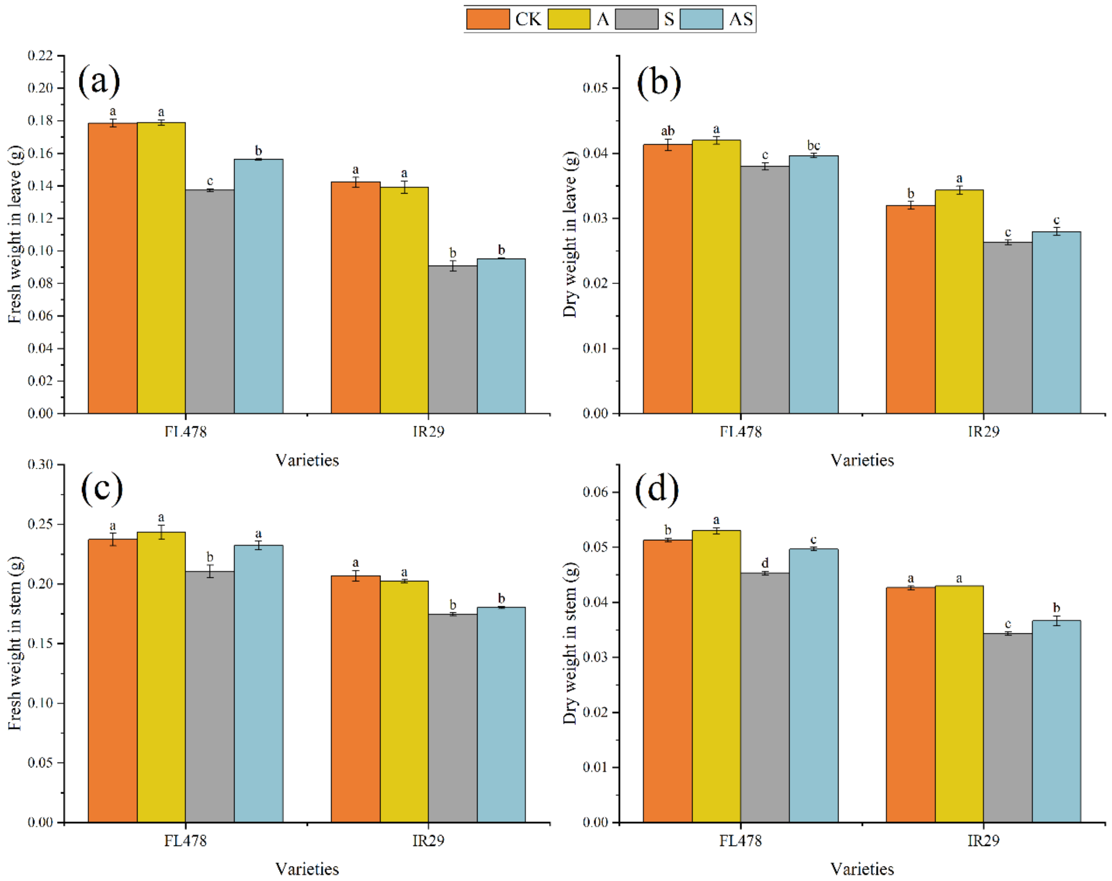

Figure S1. Effect of Alginate oligosaccharides and Salt on Major Components of Rice Cell Wall Fresh leaf weight (a), Leaf dry weight (b), Stem fresh weight (c), Stem dry weight (d). Control (CK), alginate oligosaccharides (A), salt (S), and alginate oligosaccharides and salt (AS). Different letters in the same variety indicate significant differences between the treatments ($p < 0.05$).

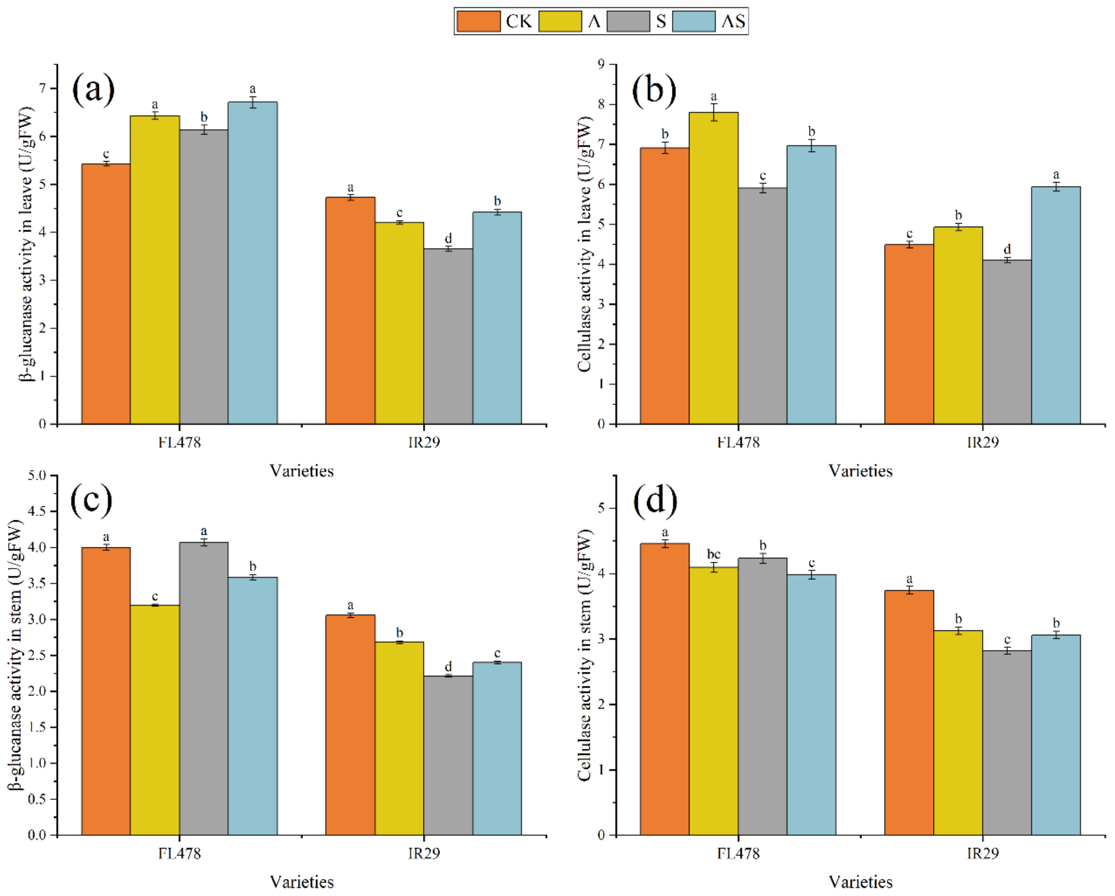

Figure S2. Effect of alginate oligosaccharides and salt on rice cellulase and β-glucanase activities. Leaf β-glucanase activity (a), Leaf cellulase activity (b), Stem β-glucanase activity (c), Stem cellulase activity (d). Control (CK), alginate oligosaccharides (A), salt (S), and alginate oligosaccharides and salt (AS). Different letters in the same variety indicate significant differences between the treatments ($p < 0.05$).

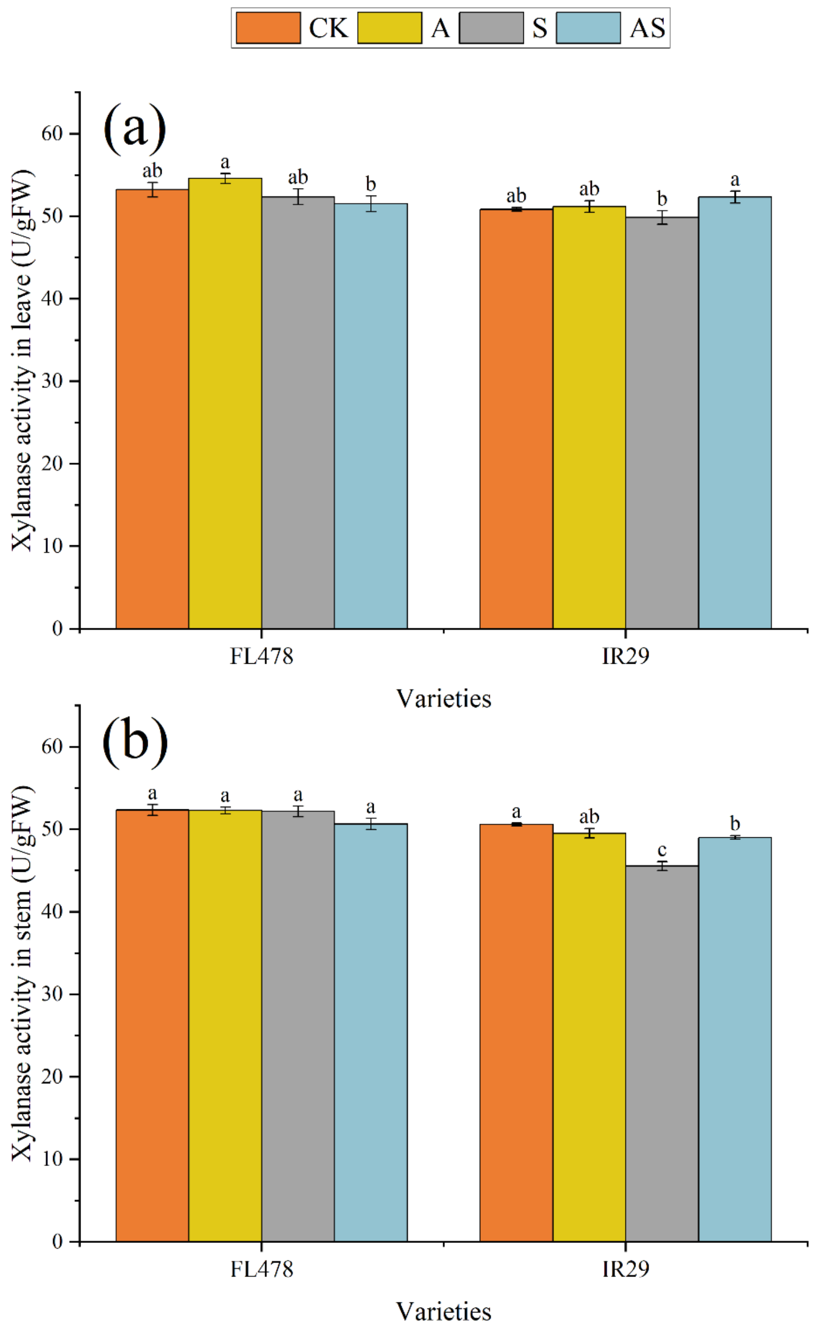

Figure S3. Effect of alginate oligosaccharide and salt on rice hemicellulose metabolism. Leaf xylanase activity (a), Stem xylanase activity (b). Control (CK), alginate oligosaccharides (A), salt (S), and alginate oligosaccharides and salt (AS). Different letters in the same variety indicate significant differences between the treatments ($p < 0.05$).

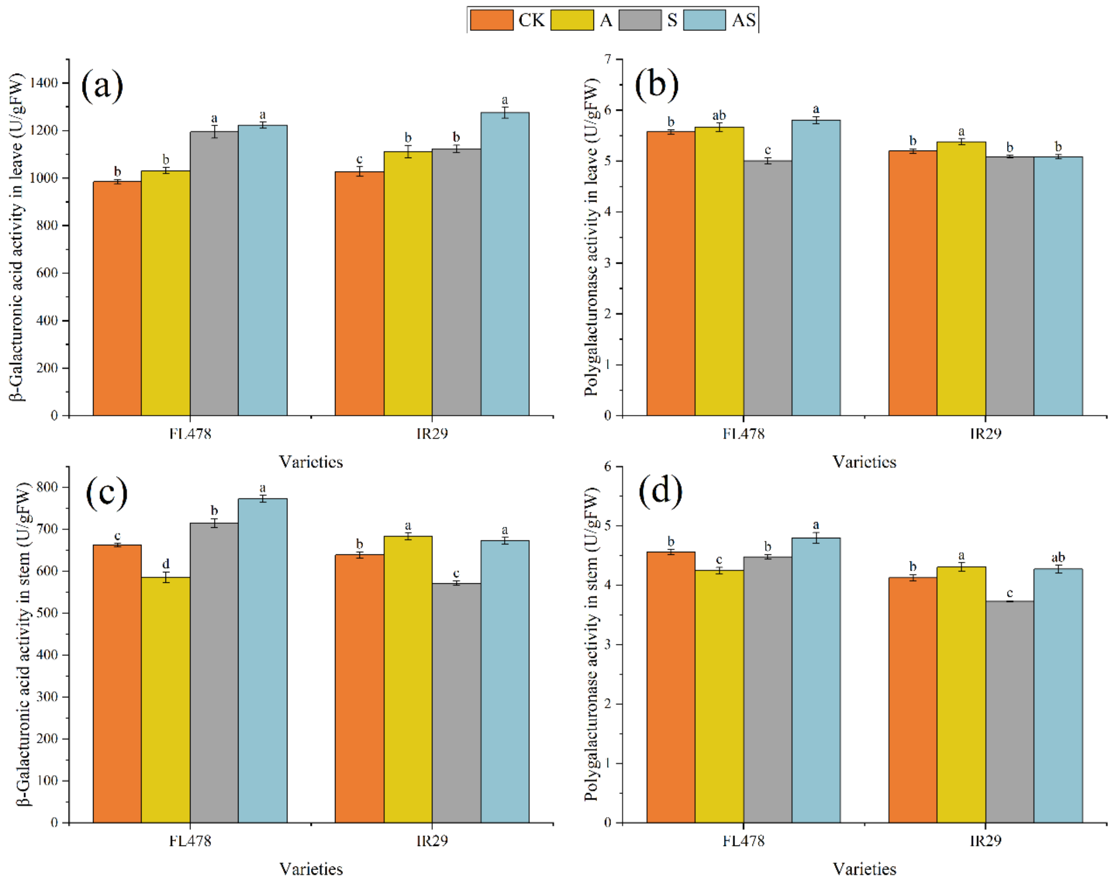

Figure S4. Effect of alginate oligosaccharides and salts on enzyme activities of rice pectin metabolism. Leaf β-galactosidase activity (a), Leaf polygalacturonase (b), Stem β-galactosidase activity (c), Stem polygalacturonase (d). Control (CK), alginate oligosaccharides (A), salt (S), and alginate oligosaccharides and salt (AS). Different letters in the same variety indicate significant differences between the treatments ($p < 0.05$).
